# Do not smile when acquiring consumer selfies for multi-attribute skin profiling and baseline deep learning evaluation

**DOI:** 10.1038/s41598-026-65514-4

**Published:** 2026-08-08

**Authors:** Dennis Hartmann, Dominik Müller, Florian Auer, Gabriele Marie Lehner, Laura Gockeln, Anna Rottenkolber, Gabriel Duttler, Julia Welzel, Frank Kramer

**Affiliations:** 1 IT-Infrastructure for Translational Medical Research, University of Augsburg, Augsburg, Germany; 2 Department of Dermatology and Allergology, University Hospital Augsburg, Augsburg, Germany; 3 Grandel the Beautyness Company, Augsburg, Germany

**Keywords:** Medical image analysis, Biomedical image classification, Computational biology and bioinformatics, Diseases, Engineering, Health care, Mathematics and computing, Medical research

## Abstract

The increasing demand for personalized skincare solutions highlights a significant gap: many consumers struggle to find suitable products without professional guidance. While the commercial potential for tailored product recommendations is vast, a key challenge remains the lack of effective methods for skin profiling via image classification. To address this challenge, this paper introduces a comprehensive benchmark dataset of 3203 standardized facial consumer selfies, annotated across eight primary cosmetic skin features. We establish a transparent baseline evaluation utilizing the open-source medical image classification framework AUCMEDI. A variety of deep learning architectures were trained to recognize and classify various skin features, including sagging skin, wrinkles, under-eye circles, redness, shine, pigment spots, acne, and pore size. The baseline model’s performance was evaluated using the mean absolute error (*e*), which appropriately accounts for the ordinal distance in cosmetic grading. Utilizing standard deep learning architectures, the baseline benchmark established promising performance in structural categories like sagging skin (*e = 1.71*) and wrinkles (*e = 1.40*). While achieving satisfactory results for under-eye circles (*e = 0.32*), redness (*e = 2.22*), and shine (*e = 0.23*), the baseline models encountered significant architectural limitations when resolving highly localized or imbalanced features such as acne (*e = 2.59*), pore size (*e = 2.57*), and pigment spots (*e = 2.20*), the latter three underperforming a trivial constant-mean predictor.

## Introduction

Deep learning algorithms, particularly convolutional neural networks, have emerged as a dominant force in medical image analysis^1–3^. Their superior performance in tasks like image classification, segmentation, and object detection has significantly advanced the field^1–3^. Medical image classification (MIC), a fundamental task involving the categorization of medical images into predefined classes (e.g., health status, tumor type), is a prime application area for these models. Accurate MIC can support clinical decision-making and improve diagnostic efficiency^4,5^. Given the critical implications of MIC outcomes, such as influencing patient prognosis, and potentially leading to misdiagnosis if inaccurate, rigorous evaluation and validation are essential to ensure reliability and clinical applicability^6^.

The demand for products and services designed to improve skin health and appearance continues to grow. Digital tools for skin analysis are playing an increasingly important role in this field^7^. These technologies enable the assessment of facial features and skin conditions, facilitating the development of personalized skincare regimens. However, a significant challenge is the lack of robust, standardized methods for automated skin profiling through image analysis. While major commercial entities (e.g., L’Oréal, Haut.AI, Perfect Corp, and Canfield) have successfully deployed multi-attribute facial assessment systems at scale, their underlying datasets, annotation protocols, and modeling methodologies remain strictly proprietary. Consequently, the academic community lacks a standardized, open-source benchmark to evaluate and advance selfie-based cosmetic profiling. This work directly addresses this gap.

To prevent conceptual misinterpretation, we explicitly clarify the intended use and regulatory framing of this system: this approach is strictly designed as an appearance-based profiling tool for cosmetic skin feature assessment and personalized skincare guidance. It is explicitly non-diagnostic and is not intended to replace professional clinical dermatological evaluation or to function as a medical device.

The primary contribution of this work is twofold: first, we present a large-scale, multi-class cosmetic dataset annotated by trained evaluators under real-world smartphone and webcam capture variations. Second, we establish a transparent baseline benchmark utilizing the AUCMEDI framework across twelve established deep learning architectures to demonstrate the non-trivial nature of this data. By openly sharing our pipeline and anonymized metadata, this work serves as a foundational benchmark for future algorithmic improvements in automated cosmetic skin profiling.

## Materials and methods

To provide a comprehensive overview, the dataset and pipeline are explained in the following sections.

### Dataset

**Fig. 1:**
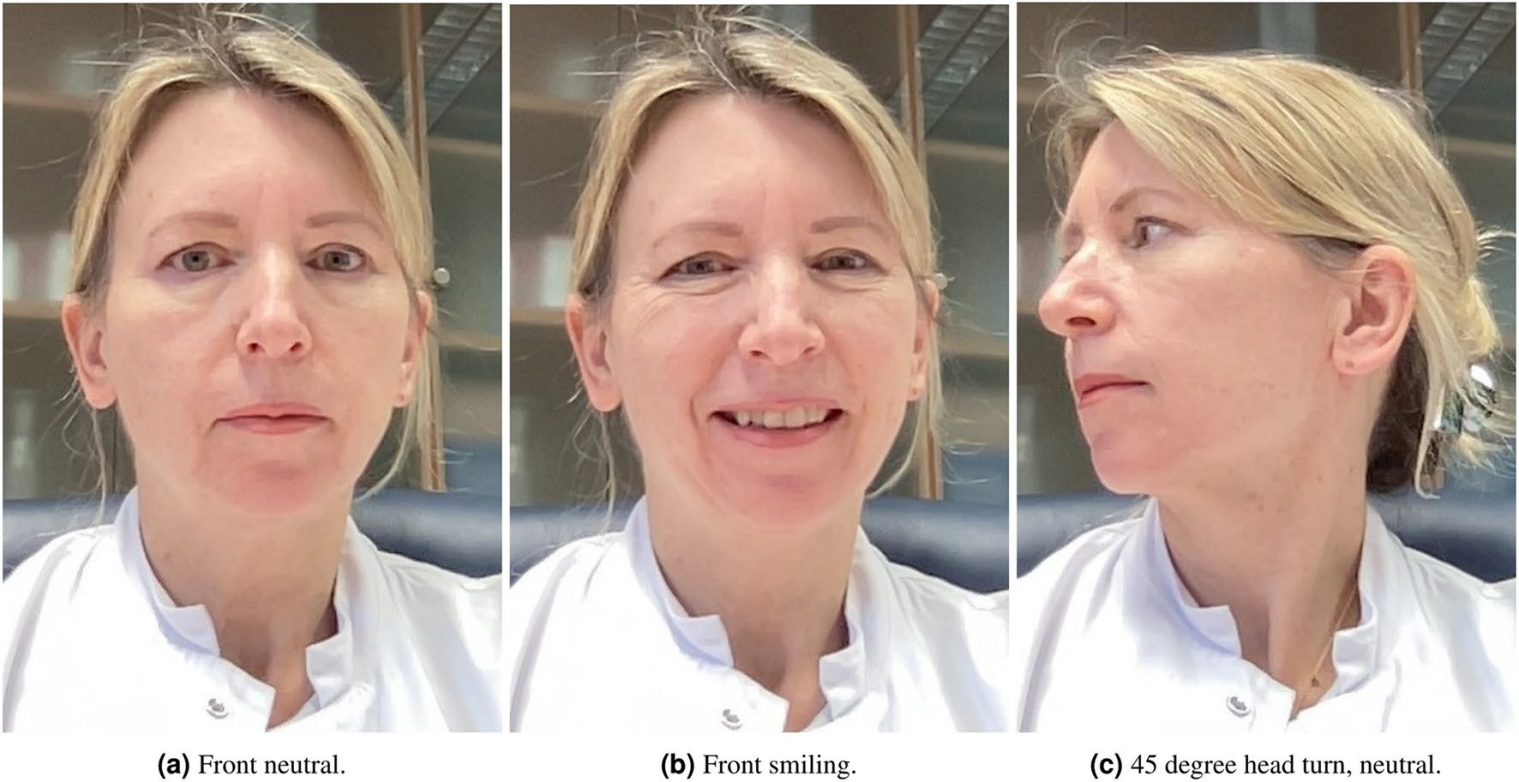
Representative images from the recordings of the 3 poses.

Photographic RGB-images of human faces were acquired between May 2023 and May 2024 through a web interface on the website of GRANDEL – The Beautyness Company. This interface was developed by drumedar Internet-Entwicklungs-GmbH. The participants for the photos were recruited through Clickworker, through newsletters, or by direct appeal. To improve consistency, a circular positioning aid was incorporated into the web interface to guide participants in aligning their heads during image acquisition. The images were therefore captured using differing technologies for image capture (e.g. webcams and smartphones with different resolutions and image processing). The images were captured in a frontal orientation with a neutral expression, a frontal orientation with a smile, and a 45-degree leftward turn with a neutral expression (as can be seen in Fig. 1.

The dataset comprises photographs of 5052 participants (3517 females and 1535 males, the latter including a subgroup of 221 bearded individuals). Since ethnic background was not self-reported, we performed a manual post-hoc annotation based on visual assessment. The dataset is predominantly Caucasian (*93.5%*, n=4725). To ensure demographic transparency, further identified groups include participants of African (120 females, 76 males), Asian (65 females, 35 males) and Middle Eastern (8 females, 23 males) descent. Of the 5052 participants, 3203 met the inclusion criteria: no duplicate, good image quality, and participant visibility.

**Fig. 2:**
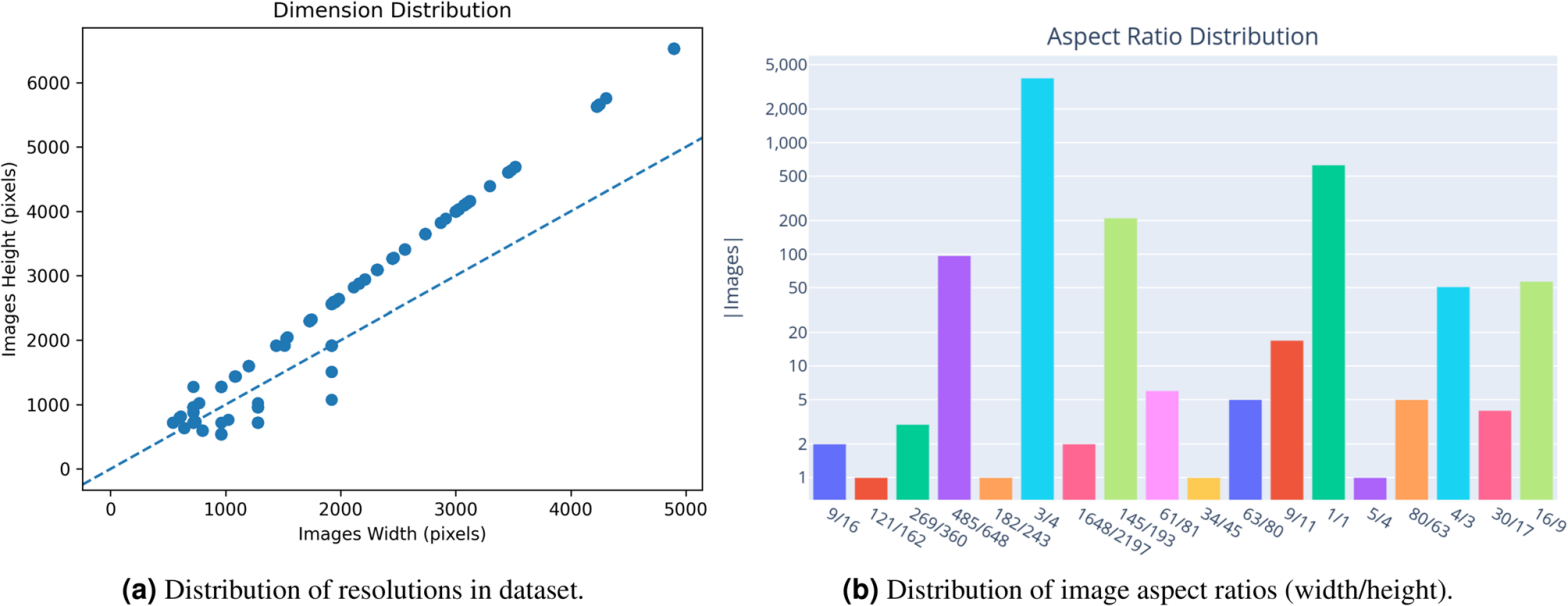
Pixel dimensions and aspect ratios of images in dataset.

Due to the use of various webcams and smartphone front cameras the images exhibit significant variability in exposure, color balance, contrast, resolution, and overall quality. Figure 2 illustrates the wide range of resolutions and ratios present in the dataset. The majority of images exhibited a relatively consistent ratio and only a small number of outliers were identified, we decided not to incorporate them into subsequent analyses, but incorporated them into the dataset.

To rigorously characterize the image quality and the actual facial signal available within the dataset, we evaluated the bounding box dimensions of the face regions using automated face detection via OpenCV’s Haar Cascade classifier across all images. Out of 3203 total images, a facial region was successfully localized in 3187 instances (99.5%), demonstrating the high consistency of consumer compliance during acquisition. While the raw photographic dimensions varied due to differing smartphone hardware, the uncompressed facial crops exhibited a substantial average resolution of *1345 × 1345* pixels (ranging from a minimum of *110 × 110* pixels to a maximum of *3932 × 3932* pixels). This high native resolution underlines the value of the dataset as a genuine resource for fine-grained skin analysis.

The annotation was performed by 5 dermatologist-trained non-professionals, which were re-instructed after regular evaluations. Each annotator scored participants, not overlapping, for all eight classes: Under-eye circles and shine were classified as binary variables (present 1 or absent 0), while acne, wrinkles, facial redness, sagging skin, pigment spots, and pore size were rated on an integer scale of 0 to 10. The rating scales of the individual classes are hereinafter referred to as subclasses.

**Fig. 3:**
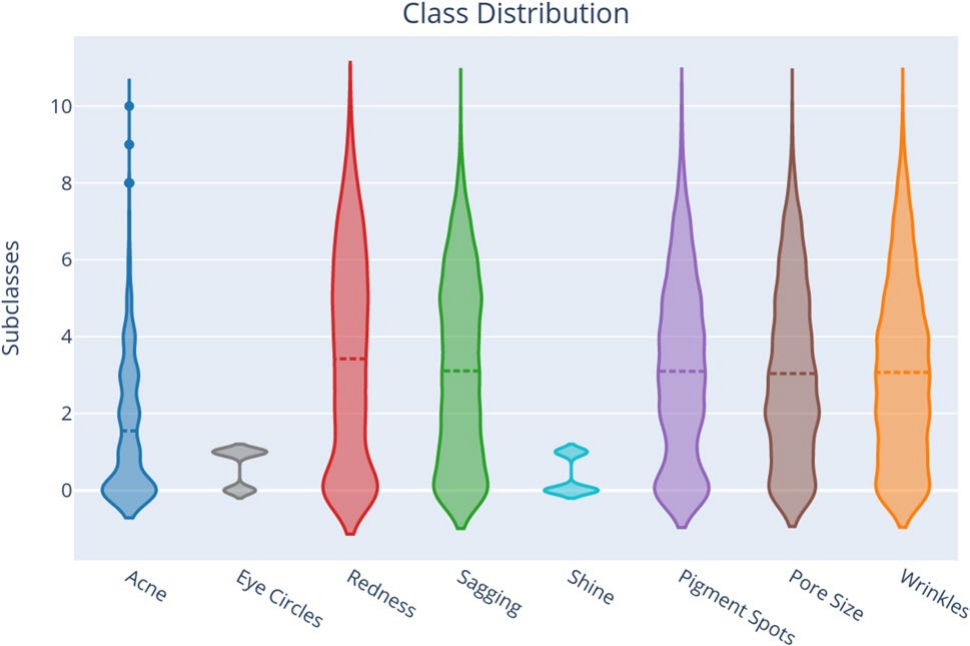
Subclass distribution of all classes.

For acne, the frequencies begin with 1308 instances in the first class and steadily decline to 1 in the final of the eleven classes. Similar trends are observed for wrinkles and pigment spots, with distributions ranging from 516 to 2 and 639 to 6, respectively. Facial redness starts with 735 instances and concludes with 16, with minor fluctuations observed in the intermediate classes. The distribution of pore size begins at 510, reaches a peak of 560 in class 2, and then declines to 2. The frequencies for sagging skin span a range from 610 to 1 across its eleven classes. In contrast, the distributions for under-eye-circles and shine are 1182 to 2021 and 2008 to 1195, respectively. The class distributions are further illustrated in Fig. 3.

For classes categorized binary we found a slight class imbalance in the dataset (shine and under-eye-circles:  37 %), which was considered acceptable for the training of the models. However, a challenge was the limited availability of participants exhibiting the extreme scorings of the classes with 11 subclasses, which led to a less uniform data distribution across the rating scale.

The dataset was built incrementally, with the model undergoing the steps, at each development stage, outlined in the next chapter.

### Pipeline

**Fig. 4:**
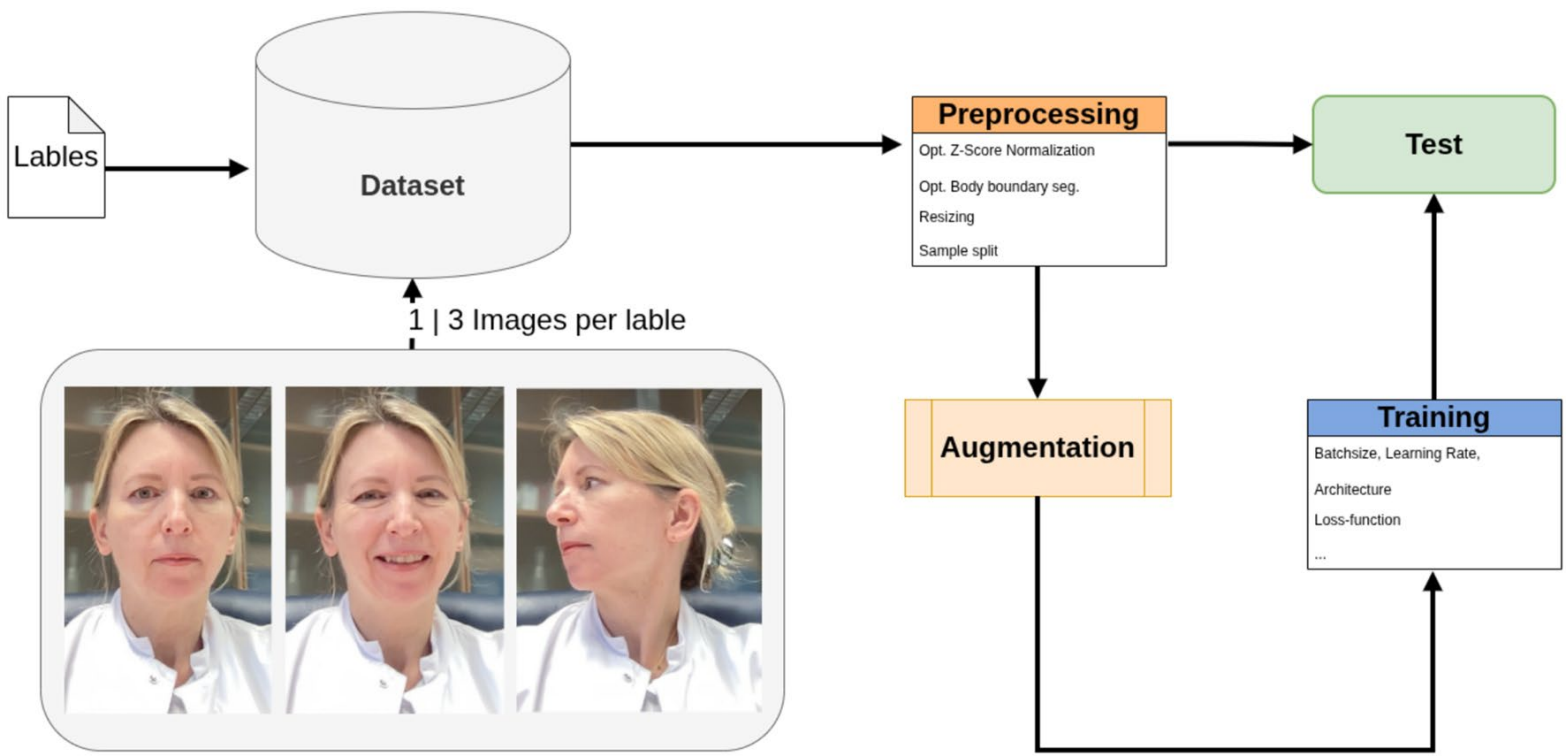
Graphical representation of the pipeline architecture.

The AUCMEDI package offers a comprehensive API library for constructing standardized, state-of-the-art image classification pipelines^8,9^. By utilizing this framework, we established a robust and easily integrable pipeline that could be seamlessly deployed. AUCMEDI facilitated image preprocessing and neural network model management, ensuring a standardized and reproducible environment for diverse infrastructures. The graphical representation of the pipeline’s structure is illustrated in Fig. 4.

#### Preprocessing

For this study, we explored four input modalities: single frontal neutral, frontal smiling, 45 degree head turned neutral face expression, and stacked images containing all three views in the third dimension. These images were split into training (70 %), validation (10 %) and testing (20 %) sets and resized via bi-linear interpolation to match the input dimensions of the neural network architectures (224 px x 224 px).

The pipeline has been enhanced to incorporate z-score normalization and the option to remove background elements. The z-score normalization was performed using the function pre-implemented in AUCMEDI, with the default parameters^8^. For background removal, the rembg package was used to create a segmented image of the human body^10^. Then, this image was cropped to the body boundaries and the background was replaced with a solid black color.

To prevent overfitting online data augmentation techniques (mirroring, rotation, scaling, elastic transformation, noise addition, brightness/contrast/gamma adjustments) have been implemented in the preprocessing step of the pipeline.

#### Training

A comprehensive set of deep learning models was employed, including DenseNet variants (121, 169, 201)^11^, ResNet variants (50, 101, 152)^12^, ResNeXt variants (50, 101)^13^, MobileNet^14^, MobileNetV2^15^, VGG16 as well as VGG19^16^, ConvNeXtBase and ConvNeXtLarge^17^. Each model was capable of using a regression- or classification head. The classification head utilizes a softmax activation function to generate a probability distribution over |subclasses of class| potential categories (i.e. 2 or 11). The regression head employs a ReLU activation function to output a single continuous value. Instead of utilizing a one-versus-all approach, a separate model was trained for each individual class.

To optimize training, we conducted experiments with various parameters like batch sizes and learning rates. The batch size was systematically increased in increments of 30, commencing with a batch size of 30 and culminating at 240. Subsequently, an exhaustive investigation was conducted to identify the optimal batch size within the range of the two most promising values. The ADAM optimizer^18^ was used in conjunction with pre-trained ImageNet^19^ weights for the corresponding selected architecture. Tested loss functions included categorical focal loss^20^, categorical cross-entropy, and mean absolute error.

A transfer learning approach was adopted, where the initial 10 epochs were dedicated to train only the head layers of the network with a fixed learning rate of $1e^{-4}$. Subsequently, all layers were fine-tuned with a dynamic learning rate that started at $1e^{-4}$ and decreased to $1e^{-7}$ over a maximum of 290 epochs. Early stopping was implemented to prevent overfitting, terminating training after 30 epochs without improvement in validation loss. A learning rate reduction factor of 0.1 was applied every 10 epochs without progress.

#### Testing and evaluation

To ensure reproducibility, a single, standardized test dataset was used to evaluate all models in this study as described. For the quality of the predictions, the standard measurement values for the quality in MIC are based on an exact prediction. The classification in this domain is subject to inherent variability due to differences in human perception. The observed deviations are within the expected range of inter-rater disagreement and are therefore considered acceptable. For a clear representation of the prediction quality, the mean absolute error *e* was used (shown in Formula 1). The smallest possible *e* is considered positive, whereas a large *e* is unfavorable. In this context, *n* describes the number of images, *p* the prediction and *a* the ground truth annotation of the class *k* of image *i*.

1 $$\begin{aligned} e_k = \frac{(\sum )^{n -1}_{i = 0} |p_{k, i} - a_{k, i}|}{n} \end{aligned}$$

However, using only the average absolute error *e* is insufficient, as severe class imbalances within the dataset can distort the performance evaluation. For instance, a naive majority-class or pure zero prediction yields a deceptively low error *e* for several primary attributes due to this distributional skew (as demonstrated in Table 1). To resolve this and to properly account for the distinct target variable scales, we expand our evaluation framework. For the binary classification targets (Shine and Under-eye Circles), performance is quantified using *e* alongside the $F_1$-score to safeguard against class distribution imbalances. For the 0 to 10 ordinal cosmetic scales, standard multi-class classification metrics are sub-optimal, as they fail to reflect distance; they penalize a minor near-miss (e.g., predicting a severity level of 5 instead of 4) identically to a catastrophic failure (e.g., predicting 10 instead of 4). Therefore, to preserve the ordinal hierarchy and evaluate the baseline models’ capacity to capture monotonic progression trends, we report Spearman’s rank correlation coefficient (*ρ*) in conjunction with *e*. The confusion matrix was primarily used for the assessment. Further details on robust classifier performance evaluation methodologies are provided in the comprehensive framework by Maier-Hein et al.^6^.

**Table 1:** e for a pure 0 classification per class, rounded to 3 decimal digits.

Class	Count	*e*
Acne	11	1.729
Facial Redness	11	3.750
Pigment spots	11	3.354
Pore size	11	3.167
Sagging skin	11	3.434
Shine	2	0.335
Under-eye circles	2	0.625
Wrinkles	11	3.312

## Results

Due to the extensive nature of our experiments, we present only the most promising model architectures and hyperparameters in this chapter. The presented models use the classification head, were trained on single frontal images with neutral facial expressions and the categorical focal loss.

To ensure a rigorous evaluation, all models were compared against a trivial constant predictor (the mean of the dataset). The models performances varied across the eight categories. For conditions measured on a scale of 0 to 10, the results were as follows: Acne achieved an $e_{model} = 2.59$ with an $e_{trivial} = [1.50]$ (ConvNeXtLarge, batch size 60), Facial Redness reached an $e_{model} = 2.22$ with an $e_{trivial} = [2.34]$ (ConvNeXtBase, batch size 126), Pigment Spots resulted in an $e_{model} = 2.20$ with an $e_{trivial} = [1.90]$ (ConvNeXtBase, batch size 150), Pore Size reached an $e_{model} = 2.57$ with an $e_{trivial} = [1.84]$ (ConvNeXtLarge, batch size 90), Sagging Skin achieved an $e_{model} = 1.71$ with an $e_{trivial} = [2.03]$ (ConvNeXtBase, batch size 25) and Wrinkles resulted in an $e_{model} = 1.40$ with an $e_{trivial} = [1.87]$ (ConvNeXtLarge, batch size 60).

**Fig. 5:**
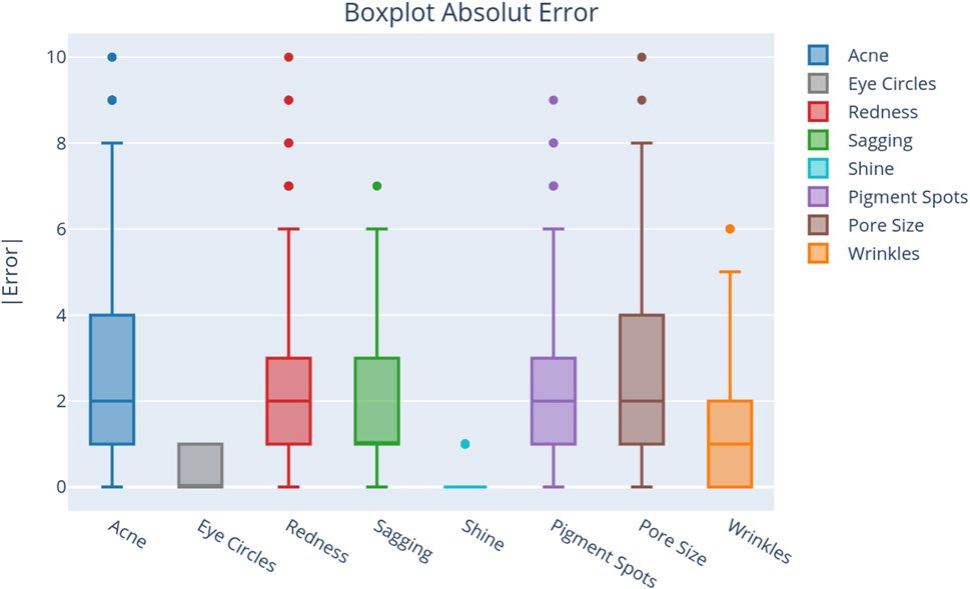
Boxplot of absolute error (|prediction - ground truth|) for all classes.

**Fig. 6:**
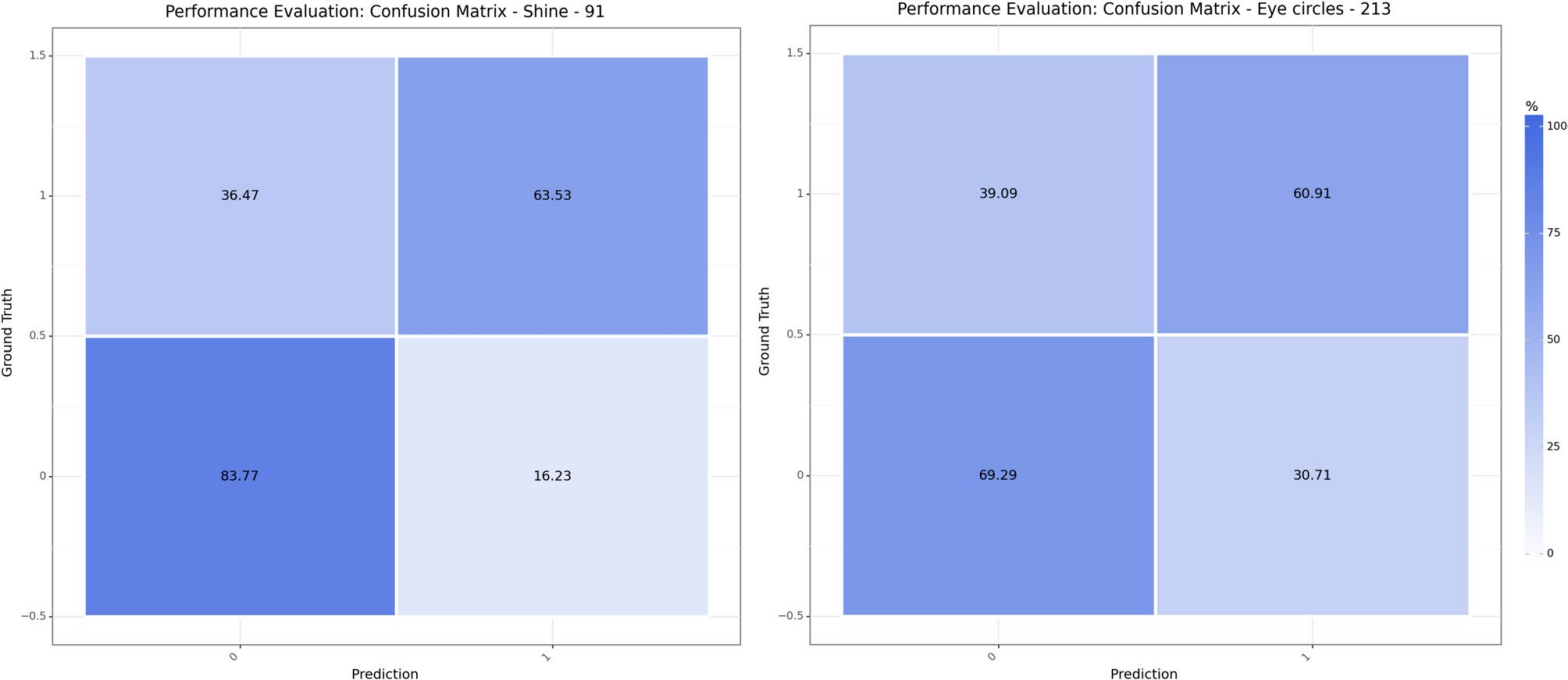
Confusion matrices of top configurations for the binary classes.

**Fig. 7:**
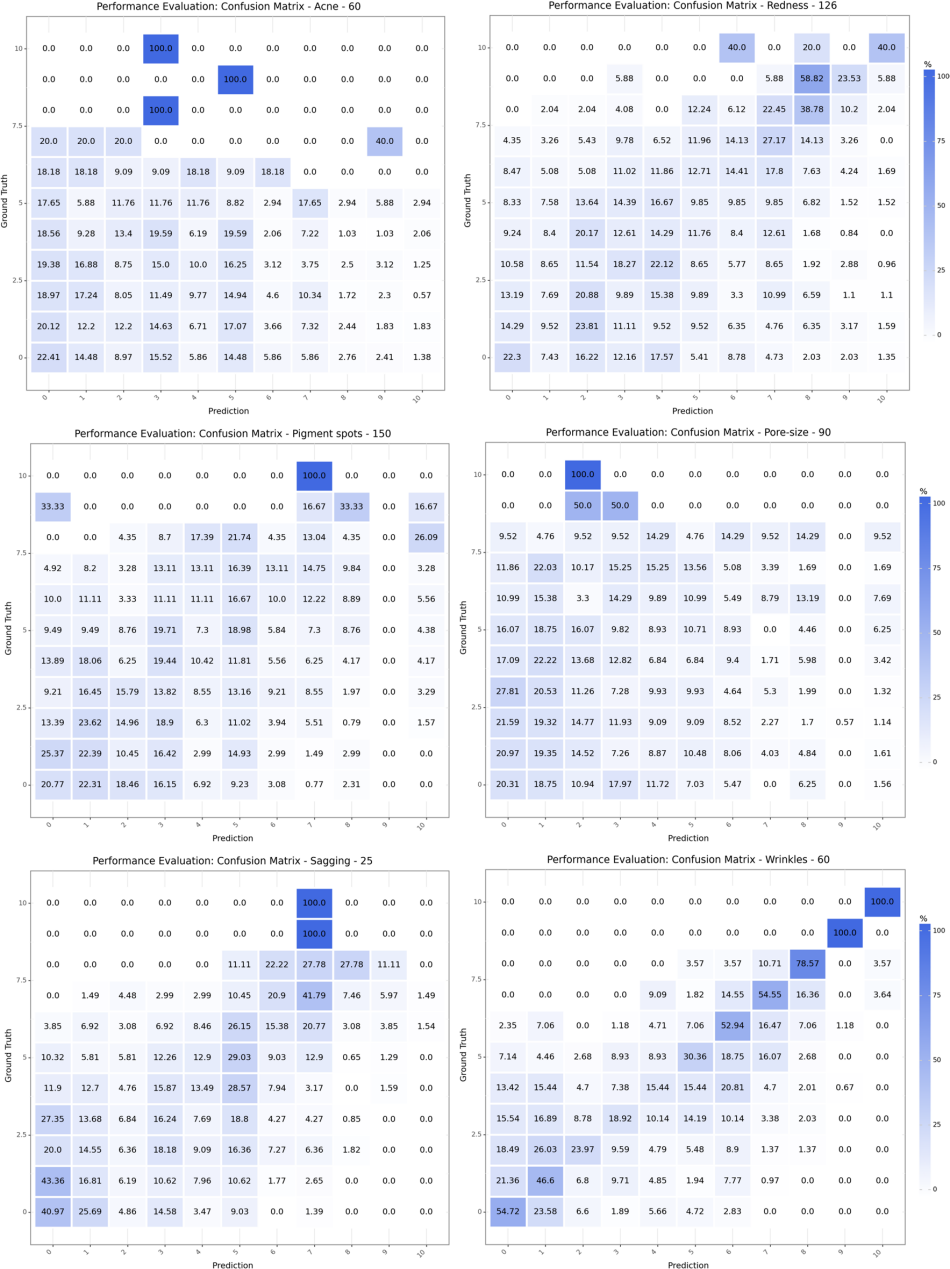
Confusion matrices of top configurations for the classes with 11 subclasses.

For conditions measured on a scale of 0 to 1, the models showed the following performance: Shine achieved an $e_{model} = 0.23$ with an $e_{trivial} = [0.37]$ (ConvNeXtBase, batch size 91) and Under-eye circles resulted in an $e_{model} = 0.32$ with an $e_{trivial} = [0.37]$ (ConvNeXtBase, batch size 213). Detailed configurations are summarized in Table 2. Visual representations of the top configurations, including boxplots and confusion matrices, are provided in Figs. 5, 6, and 7.

To evaluate the impact of facial expression and head pose on model performance, we conducted a comparative analysis across three distinct image modalities: front neutral, front smiling, and a 45-degree neutral head turn. For this analysis, we applied the model configurations optimized on the front neutral dataset. The comparative measures are detailed in Table 3. To formally test whether the observed differences between modalities are statistically robust rather than incidental, we additionally performed a pre-specified, Holm-corrected paired comparison (Wilcoxon signed-rank test for the six ordinal classes; McNemar’s test for the two binary classes) on a per-sample basis, together with matched-pairs rank-biserial correlation as effect size. Results are reported in Table 4.

To assess whether model performance varies systematically with skin tone, we computed an objective, automated skin tone proxy for all test-set images using the Individual Typology Angle (ITA), derived from CIELAB color values sampled from cheek regions detected via automated facial landmark localization. Test images were classified into six standard ITA categories (very light to dark). This approach was chosen over manual ethnicity re-annotation because it is fully reproducible and avoids the inter-rater reliability concerns inherent to subjective, post-hoc visual labeling (see Ground Truth Determination).

*e*, F1, and Spearman’s *ρ* for all eight classes are reported in Table 5 (Acne, Under-eye circles, Wrinkles, Facial redness, Shine, Sagging skin) and Table 6 (Pigment spots, Pore-size). Across all eight classes, no consistent monotonic performance degradation toward darker skin tones was observed descriptively; for several classes (Pigment Spots, Sagging Skin, Facial Redness), the ’dark’ subgroup (n=28–47) showed comparable or better e than the sample average. A pre-specified permutation test (10,000 permutations per class) compared the ’dark’ ITA subgroup against the remaining test set for all eight classes, using e as the comparison metric for ordinal classes and F1-score for binary classes. After Holm correction for multiple comparisons across the eight tests, no class showed a statistically significant difference (all $p_{adj} \ge 0.49$; Table 7). The numerically largest, though still non-significant, difference was observed for Pigment Spots (observed difference=0.580, *p*=0.062, $p_{adj}$=0.494), where the ’dark’ subgroup (n=34) showed better performance than the remaining test set (e=1.65 vs. 2.23), a direction opposite to a typical bias concern. Full stratified metrics (*e*/F1/*ρ* per ITA category) are reported in Tables 5 and 6, and complete per-class permutation test results in Table 7.

**Table 2:** Configurations and scores of top neural networks $e_{model}$, mean dataset predictor $e_{trivial}$, F1 score as well as Spearman’s rank correlation coefficient *ρ*.

Class	Count	Architecture	Batchsize	$e_{model}$	$e_{trivial}$	F1	*ρ*
Acne	11	ConvNeXtLarge	60	2.59	1.50	0.078	0.128
Facial redness	11	ConvNeXtBase	126	2.22	2.34	0.188	0.191
Pigment spots	11	ConvNeXtBase	150	2.20	1.90	0.114	0.194
Pore-size	11	ConvNeXtLarge	90	2.57	1.84	0.087	0.128
Sagging skin	11	ConvNeXtBase	25	1.71	2.03	0.180	0.253
Shine	2	ConvNeXtBase	91	0.23	0.37	0.740	0.584
Under-eye circles	2	ConvNeXtBase	213	0.32	0.37	0.643	0.383
Wrinkles	11	ConvNeXtLarge	60	1.40	1.87	0.412	0.335

**Table 3:** Mean absolute error *e*, F1 score as well as Spearman’s rank correlation *ρ* coefficient for the eight classes across three modalities: front neutral, front smiling and 45 degree head turn neutral.

	Front neutral			Front smiling			45 degree neutral		
Class	*e*	F1	*ρ*	*e*	F1	*ρ*	*e*	F1	*ρ*
Acne	2.59	0.078	0.128	3.13	0.053	0.140	2.69	0.077	0.192
Facial redness	2.22	0.188	0.191	3.36	0.102	0.085	3.65	0.097	0.092
Pigment spots	2.20	0.114	0.194	5.12	0.031	-0.002*\dag*	5.31	0.037	-0.029*\dag*
Pore-size	2.57	0.087	0.128	2.78	0.077	0.131	2.69	0.088	0.110
Sagging skin	1.71	0.180	0.253	1.99	0.131	0.239	2.03	0.159	0.251
Shine	0.23	0740	0.584	0.43	0.568	0.157	0.43	0.561	0.156
Under-eye circles	0.32	0.643	0.383	0.40	0.572	0.210	0.57	0.402	-0.129
Wrinkles	1.40	0.412	0.335	1.92	0.128	0.232	1.74	0.134	0.225

Values marked with *\dag* are not statistically significant (*p > 0.05*).

**Table 4:** Statistical comparison of front-neutral vs. front-smiling and front-neutral vs. 45-degree head turn, per class.

Class	Neutral vs. Smiling		Neutral vs. 45-degree	
	$p_{adj}$	*r*	$p_{adj}$	*r*
Acne	0.121	0.762	1.000	0.710
Under-eye circles	1.000	0.015	0.011	0.212
Wrinkles	0.0099	0.877	0.0099	0.866
Facial redness	2.6e-05	0.859	8.4e-04	0.868
Shine	0.387	0.109	0.121	0.148
Sagging skin	0.487	0.829	0.473	0.799
Pigment spots	1.5e-15	0.927	4.1e-14	0.920
Pore-size	1.000	0.695	1.000	0.655

$p_{adj}$ = Holm-corrected *p*-value across all 16 tests; *r* = matched-pairs rank-biserial correlation (Wilcoxon) or difference in error rates (McNemar).

**Table 5:** ITA-stratified performance across the test set (front-neutral modality).

ITA Cat.	Acne		Eye circ.		Wrinkles		Redness		Shine		Sagging	
	n	*e*(*ρ*)	n	*e*(F1)	n	*e*(*ρ*)	n	*e*(*ρ*)	n	*e*(F1)	n	*e*(*ρ*)
Very light	146	2.45(-0.03)	129	0.38(0.66)	134	1.41(0.62)	138	2.07(0.37)	124	0.20(0.63)	123	1.72(0.58)
Light	195	2.74(0.02)	178	0.35(0.69)	181	1.35(0.64)	208	2.14(0.36)	205	0.20(0.68)	219	1.69(0.58)
Intermediate	193	2.75(0.07)	206	0.37(0.70)	212	1.52(0.60)	180	2.44(0.21)	224	0.24(0.62)	207	1.86(0.49)
Tan	225	2.44(0.03)	218	0.30(0.77)	214	1.42(0.66)	198	2.24(0.36)	209	0.25(0.63)	218	1.63(0.63)
Brown	151	2.62(0.09)	170	0.28(0.81)	160	1.27(0.71)	171	2.22(0.36)	173	0.23(0.71)	171	1.73(0.52)
Dark	28	2.29(0.05)	37	0.35(0.75)	37	1.49(0.61)	43	1.93(0.49)	47	0.30(0.56)	44	1.43(0.47)
Overall	938	2.59(0.03)	938	0.33(0.73)	938	1.40(0.65)	938	2.21(0.38)	982	0.23(0.65)	982	1.71(0.57)

MAE reported for all classes; F1 for binary classes (Under-eye circles, Shine); Spearman’s *ρ* for ordinal classes. n varies slightly by class due to per-class train/test splitting.

**Table 6:** ITA-stratified performance for Pigment Spots and Pore-size (front-neutral modality).

ITA Category	Pigment Spots		Pore-size	
	n	*e* (*ρ*)	n	*e* (*ρ*)
Very light	143	2.05 (0.42)	147	2.29 (0.11)
Light	191	2.32 (0.27)	200	2.63 (0.05)
Intermediate	191	2.29 (0.31)	207	2.43 (0.14)
Tan	209	2.09 (0.34)	201	2.71 (0.11)
Brown	170	2.36 (0.27)	187	2.64 (0.05)
Dark	34	1.65 (0.48)	40	3.00 (0.12)
Overall	938	2.21 (0.33)	982	2.57 (0.12)

MAE and Spearman’s *ρ* reported per ITA category.

**Table 7:** Permutation test results (10,000 permutations) comparing the ’dark’ ITA subgroup against the remaining test set, per class.

Class	Metric	n (dark)	Diff (Rest−dark)	*p*	$p_{adj}$
Pigment spots	*e*	34	0.580	0.0617	0.494
Pore-size	*e*	40	-0.451	0.1453	1.000
Sagging skin	*e*	44	0.291	0.2138	1.000
Facial redness	*e*	43	0.299	0.3333	1.000
Shine	F1	47	0.092	0.3907	1.000
Acne	*e*	28	0.318	0.4453	1.000
Wrinkles	*e*	37	-0.086	0.7296	1.000
Under-eye circles	F1	37	-0.022	0.7879	1.000

$p_{adj}$ = Holm-corrected *p*-value across all 8 tests.

## Discussion

This paper introduces a novel cosmetic benchmark dataset evaluated via a transparent deep learning baseline utilizing frontal selfie images as input. A specialized neural network is trained for each target skin condition, enabling automated classification with varying levels of performance across all categories.

The underlying task inherently represents an ordinal regression problem. However, preliminary experiments utilizing standard regression heads combined with Mean Absolute Error (MAE) or Mean Squared Error (MSE) loss functions failed to yield usable results. Due to the severe class imbalances across all scales, the regression models rapidly collapsed toward predicting the global dataset mean to minimize the loss, failing to capture any conditional variance. To overcome this optimization failure, a classification head utilizing a softmax activation function was deployed. When paired with Categorical Focal Loss, this approach successfully forced the networks to establish distinct decision boundaries between individual severity bins, yielding stable convergence and significantly better optimization behavior. Models trained on 3D or stacked data did not yield satisfactory performance. Additionally, the categorical cross-entropy and mean squared error loss functions proved ineffective for our task. The application of a Z-score filter and background removal techniques resulted in a decline in model predictive quality. Interestingly, visual inspection confirmed that the rembg package performed exceptionally well at cleanly isolating the facial region from the background. However, the subsequent drop in classification performance indicates that removing the background context unexpectedly altered the network’s feature optimization or disrupted the global contrast balance required by the standard architectures. While background removal exhibited marginal improvements for a subset of classes, it overall led to a reduction in performance across the majority of categories. We note that this finding should be interpreted as specific to the rembg-based segmentation approach used here, rather than as a general statement about background removal for facial attribute classification; a systematic comparison against dedicated face-parsing tools (e.g., landmark-based cropping) remains an open direction for future work on this pipeline.

While our initial protocol acquired three modalities (frontal neutral, frontal smiling, and $45^\circ$ turned), preliminary qualitative inspection indicated that facial expressions confounded feature extraction for several classes. Specifically, smiling was expected to induce transient structural alterations (e.g., accentuating nasolabial folds and altering pore geometry via skin tension). Consequently, frontal neutral images were selected as the primary model input to ensure structural consistency, a methodological choice reflected in our title and formally validated by the statistical comparison below.

A pre-specified, Holm-corrected statistical comparison (Table 4) confirmed statistically significant and practically large effects (rank-biserial r=0.86–0.93) for three classes, Wrinkles, Facial Redness, and Pigment Spots, where front-neutral images yielded significantly lower error than both alternative modalities (all $p_{adj} < 0.01$). For Under-eye Circles, a significant but substantially smaller effect ($p_{adj}=0.011, r=0.21$) was observed specifically for the 45-degree comparison, consistent with the localized, shadow-dependent nature of this feature under head rotation, while no difference was found for the smiling comparison. For the remaining four classes (Acne, Shine, Sagging Skin, Pore-size), no statistically robust difference survived correction for multiple comparisons, despite comparable numerical trends. These results provide quantitative, effect-size-calibrated support for the acquisition protocol’s emphasis on neutral frontal expression, while indicating that the magnitude and specificity of the modality effect is class-dependent rather than uniform.

While our baseline architectures outperformed the trivial baseline in macro-structural categories (skin shine, under-eye circles, wrinkles, sagging skin, and facial redness), they underperformed the constant predictor on highly imbalanced and localized features, specifically acne severity, pigment spots, and pore size. The most notable achievements were observed in classifying global attributes, whereas resolving micro-features to the desired extent remained a significant challenge due to the inherent complexity of quantifying localized lesions and the unconstrained nature of consumer-acquired imagery.

Existing research on automated skin profiling is often limited in scope. Although various studies deal with specific skin problems such as acne or dark circles, they usually focus on a single category with a limited number of subclasses^7,21–28^. To our knowledge, no comparable academic work exists that covers the broad, multidimensional range of values we have chosen. Moreover, commercial entities, such as Douglas or Garnier, offer publicly accessible tools for such analyzes, yet transparency regarding the underlying methodologies and performance measures is lacking.

The sub-baseline performance in acne and pore size classification highlights a key characteristic of the dataset: extreme class imbalance and the highly localized nature of these features. Since the majority of participants exhibited low severity scores, a trivial predictor achieving a low absolute error (*e*) sets a high mathematical threshold. Standard CNN architectures trained on global facial images struggle to resolve microscopic or highly sparse features like pores and acne lesions without explicit attention mechanisms. Crucially, this poor performance on fine-grained attributes is a predictable consequence of spatial information loss during preprocessing. While an automated face region analysis across the dataset confirms that the uncompressed native facial crops provide a dense spatial signal–averaging *1345 × 1345* pixels (ranging from *110 × 110* to *3932 × 3932* pixels)–the mandatory downsampling to a global *224 × 224* pixel input layer required by standard deep learning backbones effectively obliterates these high-frequency textures. Micro-features are compressed into sub-pixel dimensions before reaching the initial convolutional layers. This structural limitation is intentionally documented here to serve as a baseline counter-example, proving that future researchers must utilize localized region-of-interest (ROI) cropping or multi-scale architectures on this dataset. This honest baseline underperformance underlines why this dataset is non-trivial and serves as a challenging benchmark for future methodological research.

### Non-homogeneous data

The diversity of skin types and lighting conditions encountered in real-world images presents a huge challenge. The non-homogeneity of the data can make it difficult for annotators or an AI to consistently identify and delineate e.g. acne lesions and pores. The effects of lighting conditions and image positioning on the appearance of skin features cannot be ignored. Shadows, reflections, and uneven illumination can distort the appearance of acne, pores, and other skin characteristics. These factors can introduce additional variability into the annotation process and make it difficult to accurately assess the severity of skin concerns. Additionally, variations in screen settings and color calibration can introduce discrepancies in color perception, further complicating the annotation process.

In terms of demographic distribution, the dataset is predominantly composed of Caucasian skin profiles. To ensure strict data privacy compliance (GDPR) and guarantee complete consumer anonymity during the decentralized image acquisition phase, self-reported demographic metadata, such as biological sex or ethnic background, was intentionally not collected, Because self-reported demographic metadata was intentionally not collected during the decentralized acquisition phase to ensure strict data privacy compliance, the phenotypic distribution described in the materials section relies on a post-hoc visual estimation by the authors. We explicitly acknowledge that such visual classifications are inherently subjective and susceptible to human observer bias or misclassification. Consequently, these demographic labels are provided solely to ensure baseline transparency regarding the dataset’s composition, and we strongly caution against using these baseline weights for universal demographic generalization without further validation.

### Skin tone stratification

The skin tone stratification (Tables 5, 6 and 7) provides no evidence of a systematic performance disparity toward darker skin tones for any of the eight classes in this dataset. After correcting for multiple comparisons across all eight pre-specified tests, no class-wise difference between the ’dark’ ITA subgroup and the remaining test set reached statistical significance, and the numerically largest observed difference (Pigment Spots) favored the ’dark’ subgroup rather than indicating degraded performance. This contrasts with patterns reported for some other automated skin analysis systems and should be interpreted cautiously: the statistical power of these tests is inherently limited by the small size of the ’dark’ subgroup (n=28 – 47) relative to the predominantly Caucasian composition of the dataset (93.5%), and an absence of a detected effect is not equivalent to a confirmed absence of disparity. We recommend that future work on this dataset prioritize collecting a larger, more demographically balanced test set to enable adequately powered fairness evaluation across all cosmetic attribute classes.

### Ground truth determination

One of the significant challenges in annotating is establishing a reliable ground truth. As shown by Lehner et al., there is a significant spread in the assessment of skin conditions even among dermatologists^29^. Due to the subjective nature of features, there can be a considerable degree of variability in annotations across different annotators. Determining precise thresholds for features like under-eye circles, shine, and pigment spots can be subjective and context-dependent. The appearance of under-eye circles can vary based on factors such as lighting, skin tone, and individual anatomy. Similarly, the level of shine that is considered excessive can be influenced by cultural norms and personal preferences. Ambiguities can make it challenging to establish consistent annotation guidelines. These inconsistencies lead to inconsistent annotation which can hinder the training of accurate models, as the algorithm may struggle to learn from these inconsistent labels.

A clear limitation of our annotation protocol is the use of an 11-point (0 – 10) ordinal scale without overlapping annotations, preventing the calculation of inter-rater reliability. While validated dermatological scales (such as IGA or Fitzpatrick) utilize fewer bins with photographic anchors, our ambitious 11-point scale was chosen to capture subtle cosmetic transitions. Given the resource constraints of utilizing non-professional, albeit trained, evaluators without overlap, label noise is undoubtedly present in the dataset. However, rather than invalidating the setup, this realistic ’noisy’ data reflects the messy reality of crowdsourced cosmetic labeling, making this benchmark an authentic testbed for robust machine learning approaches that can handle label noise.

### Applicability in consumer-facing scenarios

The clinical utility of medical image classification demands strict precision to avoid diagnostic errors^6^. However, in consumer-facing cosmetic guidance, the tolerance for misclassification operates under a different paradigm. This operational difference directly dictates our multi-dimensional evaluation methodology. For the binary classification targets (Skin Shine and Under-eye Circles), performance is quantified using the *e* alongside the $F_1$-score to safeguard against class distribution imbalances, yielding robust baselines for skin shine (*e = 0.23*, $F_1 = 0.740$, *ρ = 0.584*) and under-eye circles (*e = 0.32*, $F_1 = 0.643$, *ρ = 0.383*).

For the 0 – 10 ordinal cosmetic scales, however, standard hard multi-class classification metrics (such as unweighted $F_1$-scores) are sub-optimal, as they fail to reflect distance; they penalize a minor near-miss (e.g., predicting a severity score of 5 instead of 4) identically to a catastrophic failure (e.g., predicting 10 instead of 4). In cosmetic skin profiling, minor deviations are practically negligible as these continuous scores are mapped onto broader categorical intervals to guide over-the-counter product selection. To account for this ordinal nature and evaluate the models’ capacity to capture monotonic progression trends, we report Spearman’s rank correlation coefficient (*ρ*) alongside the *e* (see Table 2). Notably, while absolute error margins fluctuate for micro-features like acne (*e* = 2.59, *ρ* = 0.128) and pore size (*e* = 2.57, *ρ* = 0.128), the low correlation coefficients for these two classes indicate that the models capture only weak monotonic trends. This underscores that the system’s practical viability for non-prescriptive, trend-based consumer skincare routines is currently strongest for macro-structural features (Wrinkles, Sagging Skin, Facial Redness) and comparatively limited for localized micro-features.

### Future work

Expanding the dataset with more diverse images would further increase the performance and generalizability of the models. The experiments with different amounts of data while creating the dataset show a tendency to improve with more data. Given the different aspect ratios present in the dataset, the data could be adjusted through an initial cropping and subsequent manual verification. This would ensure that no distortions are introduced during the resizing process. Given the continuous nature of skin condition severity, a regression-based neural network is theoretically more suitable for this task than a classification-based approach. Experiments with different regression head architectures and possibly additional layers could further improve the predictions compared to the classification head. Quantifiable skin conditions, such as acne or pigment spots, are well-suited for segmentation analysis. By segmenting these conditions and counting the quantity, we can potentially achieve more precise and accurate results compared to our classification approach in this paper.

## Conclusion

This research focuses on the creation of a benchmark dataset for the automated classification of eight key skin conditions, acne, facial redness, pigmentation, pore size, sagging skin, wrinkles, shine, and dark circles, using facial selfies as input. Recognizing the inherent subjectivity of human perception and the influence of external factors like lighting and image quality, we aim to establish a standardized approach for evaluating skin health under real-world conditions.

Our baseline evaluation indicates promising outcomes in classifying certain skin concerns, particularly sagging skin and wrinkles, demonstrating the immediate utility of the benchmark dataset. While we exhibited reasonable performance in classifying under-eye circles, redness and shine, the effectiveness of standard architectures in classifying acne, pore size and pigment spots was limited. However, more research is required to achieve consistently high predictive performance across all categories. Our neural network models, when integrated with a self-assessment questionnaire, offer a valuable and data-driven approach for self-assessment of skin conditions. This hybrid method enables the provision of tailored skincare recommendations based on dermatological standards. The developed system, designated as ’S[KI]NAssistant’, is accessible via the following address: http://grandel.de/Hautanalyse. Our work is intended to help make it easier to create or improve such tools.

## Data Availability

The photographic dataset analyzed in this study contains high-resolution facial images (consumer selfies), which constitute sensitive biometric data under the European General Data Protection Regulation (GDPR) and the German Federal Data Protection Act (BDSG). Because sufficient de-identification of full-facial images is technically impossible without destroying the peripheral skin areas required for dermatological analysis, public hosting or open distribution of the raw images is strictly prohibited due to institutional ethics and privacy frameworks. To ensure scientific reproducibility and transparency, we provide a multi-layered data access pathway: 1. Open-Source Pipeline: The entire technical pipeline, preprocessing scripts, and specific training configurations utilizing the AUCMEDI framework are publicly available on GitHub (https://github.com/frankkramer-lab/GRANDAID). 2. Data on Request: Researchers seeking access to the underlying metadata structures for academic replication may contact the corresponding author upon reasonable request and subject to local data protection agreements.

## References

[1] Litjens, G. et al. A survey on deep learning in medical image analysis. *Med. Image Anal.* **42**, 60–88. 10.1016/j.media.2017.07.005 (2017). 28778026 10.1016/j.media.2017.07.005

[2] Lee, K., Zung, J., Li, P. H., Jain, V. & Seung, H. Superhuman Accuracy on the SNEMI3D Connectomics Challenge (2017).

[3] Shen, D., Wu, G. & Suk, H.-I. Deep learning in medical image analysis. *Annu. Rev. Biomed. Eng.* **19**, 221–248. 10.1146/annurev-bioeng-071516-044442 (2017). 28301734 10.1146/annurev-bioeng-071516-044442 PMC5479722

[4] Müller, D. et al. DeepGleason: A System for Automated Gleason Grading of Prostate Cancer using Deep Neural Networks. 10.48550/arXiv.2403.16678 (2024).

[5] Kumar, R., Kumbharkar, P., Vanam, S. & Sharma, S. Medical images classification using deep learning: A survey. *Multimed. Tools Appl.* **83**, 19683–19728. 10.1007/s11042-023-15576-7 (2024).

[6] Maier-Hein, L. et al. Metrics reloaded: Recommendations for image analysis validation. 10.48550/arXiv.2206.01653 (2024). 10.1038/s41592-023-02151-z PMC11182665 38347141

[7] Vatiwutipong, P., Vachmanus, S., Noraset, T. & Tuarob, S. Artificial intelligence in cosmetic dermatology: A systematic literature review. *IEEE Access* **11**, 71407–71425. 10.1109/ACCESS.2023.3295001 (2023) (**Conference Name: IEEE Access.**).

[8] Müller, D. AUCMEDI: A framework for Automated Classification of Medical Images. 10.5281/zenodo.6633540 (2022).

[9] Mayer, S., Müller, D. & Kramer, F. Standardized Medical Image Classification across Medical Disciplines. 10.48550/arXiv.2210.11091 (2022).

[10] Gatis, D. rembg Original-date: 2020-08-10T14:38:24Z. (2025).

[11] Huang, G., Liu, Z., Maaten, L. V. D. & Weinberger, K. Q. Densely Connected Convolutional Networks. 10.48550/arXiv.1608.06993 (2018).

[12] He, K., Zhang, X., Ren, S. & Sun, J. Deep Residual Learning for Image Recognition. 10.48550/arXiv.1512.03385 (2015).

[13] Xie, S., Girshick, R., Dollár, P., Tu, Z. & He, K. Aggregated Residual Transformations for Deep Neural Networks. 10.48550/arXiv.1611.05431 (2017).

[14] Howard, A. G. et al. MobileNets: Efficient Convolutional Neural Networks for Mobile Vision Applications. 10.48550/arXiv.1704.04861 (2017).

[15] Sandler, M., Howard, A., Zhu, M., Zhmoginov, A. & Chen, L.-C. MobileNetV2: Inverted Residuals and Linear Bottlenecks. 10.48550/arXiv.1801.04381ArXiv:1801.04381 (2019).

[16] Simonyan, K. & Zisserman, A. Very Deep Convolutional Networks for Large-Scale Image Recognition. 10.48550/arXiv.1409.1556 (2015).

[17] Liu, Z. et al. A ConvNet for the 2020s. 10.48550/arXiv.2201.03545 (2022).

[18] Kingma, D. P. & Ba, J. Adam: A Method for Stochastic Optimization. 10.48550/arXiv.1412.6980ArXiv:1412.6980 (2017).

[19] Deng, J. et al. ImageNet: A large-scale hierarchical image database. In *2009 IEEE Conference on Computer Vision and Pattern Recognition*, 248–255. 10.1109/CVPR.2009.5206848 (2009).

[20] Lin, T.-Y., Goyal, P., Girshick, R., He, K. & Dollár, P. Focal Loss for Dense Object Detection. 10.48550/arXiv.1708.02002 (2018). 10.1109/TPAMI.2018.2858826 30040631

[21] Chang, T.-R. & Tsai, M.-Y. Classifying conditions of speckle and wrinkle on the human face: A deep learning approach. *Electronics* **11**, 3623. 10.3390/electronics11213623**21** (2022).

[22] Zhao, T., Zhang, H. & Spoelstra, J. A Computer Vision Application for Assessing Facial Acne Severity from Selfie Images. 10.48550/arXiv.1907.07901 (2019).

[23] Zheng, Q. et al. Automatic Facial Skin Feature Detection for Everyone. 10.48550/arXiv.2203.16056 (2022).

[24] Quattrini, A., Boër, C., Leidi, T. & Paydar, R. A deep learning-based facial acne classification system. *Clin. Cosmet. Investig. Dermatol.* **15**, 851–857. 10.2147/CCID.S360450https://www.tandfonline.com/doi/pdf/10.2147/CCID.S360450 (2022). 35585864 10.2147/CCID.S360450 PMC9109724

[25] Draelos, R. L., Kesty, C. E. & Kesty, K. R. Artificial intelligence predicts Fitzpatrick skin type, pigmentation, redness, and wrinkle severity from color photographs of the face. *J. Cosmet. Dermatol.* **24**, e70050. 10.1111/jocd.70050https://onlinelibrary.wiley.com/doi/pdf/10.1111/jocd.70050 (2025). 40135957 10.1111/jocd.70050 PMC11938992

[26] Yoon, H., Kim, S., Lee, J. & Yoo, S. Deep-learning-based morphological feature segmentation for facial skin image analysis. *Diagnostics* **13**, 1894. 10.3390/diagnostics13111894 (2023). 37296746 10.3390/diagnostics13111894 PMC10252983

[27] Kim, M. & Song, M. H. High performing facial skin problem diagnosis with enhanced mask R-CNN and super resolution GAN. *Appl. Sci.* **13**, 989. 10.3390/app13020989 (2023).

[28] Li, T.-H. et al. Artificial intelligence analysis of over a million Chinese men and women reveals level of dark circle in the facial skin aging process. *Skin Res. Technol.* **29**, e13492. 10.1111/srt.13492 (2023) https://onlinelibrary.wiley.com/doi/pdf/10.1111/srt.13492. 38009029 10.1111/srt.13492 PMC10603312

[29] Lehner, G. M. et al. Differences in the annotation between facial images and videos for training an artificial intelligence for skin type determination. *Skin Res. Technol. Off. J. Int. Soc. Bioeng. Skin (ISBS) Int. Soc. Digital Imaging Skin (ISDIS) [and] Int. Soc. Skin Imaging (ISSI)* **30**, e13632. 10.1111/srt.13632 (2024). 10.1111/srt.13632 PMC10895547 38407411

